# Hepatic Differentiation of Human Induced Pluripotent Stem Cells in a Perfused Three-Dimensional Multicompartment Bioreactor

**DOI:** 10.1089/biores.2016.0027

**Published:** 2016-08-01

**Authors:** Nora Freyer, Fanny Knöspel, Nadja Strahl, Leila Amini, Petra Schrade, Sebastian Bachmann, Georg Damm, Daniel Seehofer, Frank Jacobs, Mario Monshouwer, Katrin Zeilinger

**Affiliations:** ^1^ Bioreactor Group, Berlin Brandenburg Center for Regenerative Therapies (BCRT), Charité—Universitätsmedizin Berlin, Berlin, Germany.; ^2^ Charité Centrum Grundlagenmedizin, Institut für Vegetative Anatomie, Charité—Universitätsmedizin Berlin, Berlin, Germany.; ^3^ Department of General-, Visceral- and Transplantation Surgery, Charité—Universitätsmedizin Berlin, Berlin, Germany.; ^4^ Department of Hepatobiliary Surgery and Visceral Transplantation, University of Leipzig, Leipzig, Germany.; ^5^ Janssen Research and Development, Beerse, Belgium.

**Keywords:** stem cells, tissue engineering

## Abstract

The hepatic differentiation of human induced pluripotent stem cells (hiPSC) holds great potential for application in regenerative medicine, pharmacological drug screening, and toxicity testing. However, full maturation of hiPSC into functional hepatocytes has not yet been achieved. In this study, we investigated the potential of a dynamic three-dimensional (3D) hollow fiber membrane bioreactor technology to improve the hepatic differentiation of hiPSC in comparison to static two-dimensional (2D) cultures. A total of 100 × 10^6^ hiPSC were seeded into each 3D bioreactor (*n* = 3). Differentiation into definitive endoderm (DE) was induced by adding activin A, Wnt3a, and sodium butyrate to the culture medium. For further maturation, hepatocyte growth factor and oncostatin M were added. The same differentiation protocol was applied to hiPSC maintained in 2D cultures. Secretion of alpha-fetoprotein (AFP), a marker for DE, was significantly (*p* < 0.05) higher in 2D cultures, while secretion of albumin, a typical characteristic for mature hepatocytes, was higher after hepatic differentiation of hiPSC in 3D bioreactors. Functional analysis of multiple cytochrome P450 (CYP) isoenzymes showed activity of CYP1A2, CYP2B6, and CYP3A4 in both groups, although at a lower level compared to primary human hepatocytes (PHH). CYP2B6 activities were significantly (*p* < 0.05) higher in 3D bioreactors compared with 2D cultures, which is in line with results from gene expression. Immunofluorescence staining showed that the majority of cells was positive for albumin, cytokeratin 18 (CK18), and hepatocyte nuclear factor 4-alpha (HNF4A) at the end of the differentiation process. In addition, cytokeratin 19 (CK19) staining revealed the formation of bile duct-like structures in 3D bioreactors similar to native liver tissue. The results indicate a better maturation of hiPSC in the 3D bioreactor system compared to 2D cultures and emphasize the potential of dynamic 3D culture systems in stem cell differentiation approaches for improved formation of differentiated tissue structures.

## Introduction

During drug development, only one out of nine compounds gets approved by the regulatory authorities, usually due to a lack of efficacy or toxic side effects.^1^ Thus, models for assessment of drug toxicity, especially hepatotoxicity, in the early phase of drug development are needed. Animal models, although indispensable in preclinical studies, are not sufficiently predictive for humans due to interspecies differences.^2^ Primary human hepatocytes (PHH) have been widely accepted as the gold standard for predictive *in vitro* studies on hepatic drug toxicity.^3^ However, PHH display a huge variation in cell function and enzyme activities because of interdonor variances,^4^ and the high demand of freshly isolated PHH is difficult to address due to the scarce availability of human liver tissue.

Human induced pluripotent stem cells (hiPSC) represent a promising cell source for the generation of human hepatocytes for studies on hepatic drug toxicity. Due to the unlimited self-renewing capacity of hiPSC, they provide the option for cell production in large amounts and at a constant quality. In addition, variances due to genetic polymorphism can be investigated by using different hiPSC lines representative of individual patient groups.^5^

Several protocols have been established to generate stem cell-derived hepatocytes from human pluripotent stem cells.^6–9^ These procedures mimic the embryonic development of the liver by adding different growth factors necessary for each developmental stage. The resulting hepatocyte-like cells (HLC) were successfully applied for *in vitro* studies on human drug exposure,^10,11^ hepatitis B and C infection,^12,13^ or malaria pathogenesis^14^ among others, and they have been shown to repopulate the livers of chimeric mice and rescue the disease phenotype in these animals.^15^ However, the HLC obtained with existing protocols still show an immature phenotype with reduced hepatic functionality when compared to PHH.^16,17^

To overcome these drawbacks, improved culture models are demanded, which address the needs of the cells in their natural environment. Several studies have shown that three-dimensional (3D) culture of PHH in natural or synthetic scaffolds supports cell–cell contacts, cell polarization, and preservation of liver functions such as cytochrome P450 (CYP) activities, albumin production, and glycogen synthesis.^18–20^ To improve oxygenation and medium exchange in hepatocyte cultures, various perfused 3D culture systems have been developed.^21–23^

In the 3D multicompartment bioreactor used in this study, the cells are maintained in a perfused 3D environment allowing for physiological signal exchange and autocrine or paracrine stimulation, close to the natural situation in the organ. We have previously shown that this 3D bioreactor system supports stable culture of PHH under serum-free conditions^24,25^ and is suitable for differentiation of human embryonic stem cells (hESC).^26,27^

Thus, we hypothesize that the usage of the 3D bioreactor system could improve the hepatic maturation and liver-specific functionality of hiPSC-derived hepatocytes compared with conventional two-dimensional (2D) cultures. The functionality of the cells upon differentiation in 2D cultures or 3D bioreactors was evaluated by measurement of typical hepatocyte products (albumin, urea) and CYP activities. Cultures were further characterized by means of immunohistochemical investigations, transmission electron microscopy (TEM), and analysis of liver-specific mRNA expression. Data from hiPSC-derived differentiated cells were compared to those from freshly isolated or 2D cultured PHH.

## Materials and Methods

### Bioreactor technology

The 3D multicompartment bioreactor consists of three independent, but interwoven hollow fiber capillary systems that serve for counter-current medium perfusion (two medium compartments). Cells are supplied with oxygen by direct membrane oxygenation through integrated gas capillaries (gas compartment), which are perfused with an air/CO_2_ mixture. Cells are cultured in the extracapillary space (cell compartment). The analytical scale bioreactors used in this study have a cell compartment volume of 2 mL. A detailed description of the technology is provided elsewhere.^28^

Bioreactors are operated in a perfusion device with two modular pump units, one for medium recirculation and one for medium feed. The bioreactor incubation chamber is heated by two heating units located inside the chamber, each consisting of a heating cartridge and a fan. A platinum measuring resistor monitors the temperature inside the chamber and software is used to set and maintain the desired temperature. Gas flow rates and gas compositions are regulated using electronically operated gas valves for air, CO_2_, and the resulting gas mixture (Vögtlin Instruments). Bioreactors, tubing systems, and perfusion devices were manufactured by Stem Cell Systems.

### Hepatic differentiation of hiPSC in 3D bioreactors or 2D cultures

The hiPSC line DF6-9-9T^29^ (WiCell Research Institute) was cultured under feeder-free conditions on Nunclon™ six-well cell culture plates (ThermoScientific Nunc™) coated with 8.68 *μ*g/cm^2^ Matrigel (growth factor reduced). Cells were expanded with the mTeSR™1 medium (StemCell Technologies) with 0.05 mg/mL gentamicin (Merck). Afterward, a total of 100 × 10^6^ hiPSC were seeded into a precoated bioreactor (8.68 *μ*g/cm^2^; Matrigel) and cultured over a total of 20 days.

Bioreactors were maintained at 37°C, the medium recirculation rate was 10 mL/min, and the feed rate was 1 mL/h. Based on daily measurements of the pH, glucose, and lactate values, CO_2_ and medium perfusion rates were adjusted, if necessary, to maintain a stable pH between 7.2 and 7.4 and sufficient glucose levels (>25 mg/dL).

After a proliferation phase of 3 days with mTeSR™1, differentiation of the cells in 3D bioreactors was induced based on the protocols described by Hay et al.^6,30,31^ for 2D cultures. In the first step, differentiation into definitive endoderm (DE) was induced by perfusion with the Roswell Park Memorial Institute (RPMI) 1640 culture medium (Merck) supplemented with 100 ng/mL activin A (Peprotech), 50 ng/mL Wnt3a (R&D Systems), 1 *μ*M sodium butyrate (Sigma-Aldrich), and 2% (v/v) B27 supplements without insulin (Life Technologies) for 3 days.

Subsequently, bioreactors were perfused over 13 days with a hepatocyte culture medium consisting of basal medium and single quots (Lonza) and 10 ng/mL hepatocyte growth factor (HGF; Peprotech) to induce differentiation of DE-cells to hepatoblasts. For further maturation to HLC, 10 ng/mL oncostatin M (Peprotech) was added during the last 4 days of differentiation.

2D cultures were performed in parallel with 3D bioreactor cultures for control, applying the same differentiation protocol with daily medium exchange.

### Metabolic parameters

The metabolic activity of the cells was assessed by daily measurement of glucose and lactate concentrations with a blood gas analyzer (ABL 700; Radiometer). Potential cell damage was detected by analyzing the release of lactate dehydrogenase (LDH) using an automated clinical chemistry analyzer (Cobas^®^ 8000; Roche Diagnostics) as well as the production of urea and the albumin precursor protein alpha-fetoprotein (AFP). Albumin secretion, as a marker for mature hepatocytes, was detected using an ELISA Quantitation kit and tetramethylbenzidine (TMB) substrate (both from Bethyl Laboratories) according to the manufacturer's instructions.

### Culture of PHH

The PHH were isolated from macroscopically healthy tissue that remained from resected human liver of patients with primary or secondary liver tumors or benign local liver diseases. Informed consent of the patients for the use of tissue for research purposes was obtained according to the ethical guidelines of the Charité—Universitätsmedizin Berlin. Part of the tissue sample was fixed in formaldehyde for immunofluorescence staining. Cell isolation was performed according to Pfeiffer et al.^32^

Hepatocytes were seeded at a density of 2.0 × 10^5^ cells/cm^2^ in six-well plates (BD Sciences) coated with rat tail collagen. Cells were cultivated using Heparmed Vito 143 supplemented with 10% fetal calf serum (PAA), 0.8 mg/mL insulin, 5 mg/L transferrin, 0.003 mg/L glucagon, 100 U/mL penicillin, and 100 *μ*g/mL streptomycin (all Merck).

### Measurement of cytochrome P450 (CYP) isoenzyme activities

Activities of the pharmacologically relevant CYP isoenzymes CYP1A2, CYP2B6, CYP2C9, and CYP3A4 were measured in (1) undifferentiated hiPSC, (2) HLC differentiated in 2D cultures or (3) 3D bioreactors, and (4) PHH cultures (24 h after seeding) serving as control. The cells were incubated with a cocktail containing phenacetin (Sigma) as a substrate for CYP1A2, bupropion (Toronto Research Chemicals) as a substrate for CYP2B6, diclofenac as a substrate for CYP2C9, and midazolam as a substrate for CYP3A4/5 (both from Sigma-Aldrich). Samples were taken at 1, 2, 4, and 6 h subsequent to substrate application. An overview of the used substrates, their final concentrations, and the corresponding CYP isoenzymes is provided in Table 1.

**Table 1.** **CYP Isoenzymes Tested and Their Corresponding Substrates with Resulting Products and Applied Concentrations**

Enzyme	Substrate	Product	Final concentration [*μ*M]	Transition reactions for analysis of probe products
CYP1A2	Phenacetin	Acetaminophen	100	152 → 110
CYP2B6	Bupropion	6-Hydroxybupropion	500	256 → 238
CYP2C9	Diclofenac	4′-Hydroxydiclofenac	25	312 → 266
CYP3A4/5	Midazolam	1′-Hydroxymidazolam	10	342 → 324

Formed metabolites (acetaminophen, 6-OH-bupropion, 4-OH-diclofenac, 1-OH-midazolam) were quantified by liquid chromatography tandem-mass spectrometry. Deuterated 1-OH-midazolam was added as an internal standard. Separation was carried out using an Acquity UPLC C18 column and eluted fractions were directly passed through a Xevo TQ-S tandem mass spectrometer (both from Waters Corp.). Acquired data were processed with Thermo Xcaliber 20 software (Thermo Scientific).

### Gene expression analysis

RNA was isolated from undifferentiated hiPSC, from HLC differentiated in 2D cultures or 3D bioreactors, and from freshly isolated PHH serving as reference cells. RNA isolation and subsequent cDNA synthesis were performed as described elsewhere.^33^ Each cDNA template was mixed with polymerase chain reaction (PCR) Master mix (Applied Biosystems) and human-specific primers and probes (TaqMan Gene Expression Assay system; Life Technologies; Table 2). Quantitative real-time PCR (qRT-PCR) was performed using a Real time cycler (Mastercycler ep Realplex 2; Eppendorf). The expression of specific genes was normalized to that of the housekeeping gene glyceraldehyde-3-phosphate dehydrogenase (GAPDH) and fold changes of expression levels were calculated with the ΔΔCt method.^34^

**Table 2.** **Applied Biosystems TaqMan Gene Expression Assays**^®^

Gene symbol	Gene name	Assay ID
*POU5F1*	POU domain, class 5, transcription factor 1	HS00999632_g1
*NANOG*	Nanog homeobox	HS02387400_g1
*SOX7*	SRY-box 7	HS00846731_s1
*SOX17*	SRY-box 17	HS00751752_s1
*AFP*	Alpha fetoprotein	HS00173490_m1
*ALB*	Albumin	HS00910225_m1
*CYP1A2*	Cytochrome P450 family 1 subfamily A member 2	HS00167927_m1
*CYP2B6*	Cytochrome P450 family 2 subfamily B member 6	HS03044634_m1
*CYP2C9*	Cytochrome P450 family 3 subfamily A member 4	HS00426397_m1
*CYP3A4*	Cytochrome P450 family 3 subfamily A member 4	HS00604506_m1
*GATA2*	GATA binding protein 2	HS00231119_m1
*NEFL*	Neurofilament, light polypeptide	HS00196245_m1
*GAPDH*	Glyceraldehyde-3-phosphate dehydrogenase	HS03929097_g1

### Immunofluorescence studies

Immunofluorescence staining was performed with antibodies listed in Table 3 as described elsewhere^33^ with following changes for 3D and tissue sections. The hollow fiber bed was excised *en-bloc*, fixed with 4% formaldehyde solution (Herbeta Arzneimittel), dehydrated, paraffinized, and cut into slides of 2.5 *μ*m thickness. Subsequently, the slides were deparaffinized, rehydrated, and subjected to antigen retrieval in a citrate buffer (pH 6.0) in a pressure cooker for 15 min. The same procedure was applied to native human liver tissue serving as a positive control. Nuclei in native human liver tissue were stained with bisBenzimide H 33342 trihydrochloride (Sigma).

**Table 3.** **Antibodies Used for Immunofluorescence Staining**

	Protein symbol	Species	Manufacturer	Cat.-No.	Final conc. [*μ*g/mL]
Primary antibody					
Alpha fetoprotein	AFP	mouse	Santa Cruz	Sc-8399	2
Albumin	ALB	mouse	Sigma	A6684	67
Cytokeratin 18	CK18	mouse	Santa Cruz	Sc-6259	2
Cytokeratin 19	CK19	rabbit	Santa Cruz	Sc-25724	2
Cytochrome P450 1A2	CYP1A2	mouse	Santa Cruz	Sc-53614	2
Cytochrome P450 2B6	CYP2B6	rabbit	Santa Cruz	Sc-67224	2
Hepatocyte nuclear factor 4 alpha	HNF4A	rabbit	Santa Cruz	Sc-8987	2
Marker of proliferation Ki-67	MKI67	mouse	BD Biosciences	556003	10
Multidrug resistance-associated protein 2	MRP2	rabbit	Sigma	M8316	9
POU domain, class 5, transcription factor 1	OCT3	rabbit	Santa Cruz	Sc-9081	2
Stage-specific embryonic antigen 4	SSEA4	mouse	R&D Systems	MAB1435	10
Tight junction protein 1	TJP1	mouse	LSBio	LS B2228	30
Secondary antibody					
Alexa Fluor^®^ 488 anti-mouse		goat	Life Technologies	A-11029	2
Alexa Fluor 594 anti-rabbit		goat	Life Technologies	A-11037	2

Final concentrations are given in *μ*g/mL.

Ki-67 positive cells were quantified in undifferentiated hiPSC, 2D cultured DE cells, and HLC from 2D cultures or 3D bioreactors with the open source image processing program ImageJ using at least 10 randomly chosen visual fields for each group.

### Transmission electron microscopy

For TEM, part of the 3D bioreactor cell compartment was fixed with 2.5% glutaraldehyde in 0.1 M sodium cacodylate buffer (both from Serva) over night, followed by postfixing in 1% OsO_4_ (Science Services) with 0.8% potassium ferrocyanide (Merck) in 0.1 M sodium cacodylate buffer for 1.5 h. Subsequently, samples were progressively dehydrated in ethanol and then embedded in Epon (Serva). Ultrathin sections were stained with uranyl acetate and Reynold's lead citrate (Merck) and microphotographs were taken using an electron microscope EM 906 (Carl Zeiss).

### Statistical evaluation

Experiments were performed in triplicates, unless stated otherwise, and results are presented as mean ± standard error of the mean.

The area under curve (AUC) was calculated for time courses of biochemical parameters and differences between culture systems were detected with a subsequent two-tailed Student's *t*-test. CYP activities of undifferentiated hiPSC were compared with those of HLC from 2D cultures or 3D bioreactors by one-way analysis of variance (ANOVA) followed by Bonferroni's multiple comparison test. Differences in gene expression between both culture systems were detected by applying an unpaired, two-tailed Student's *t*-test. PHH were compared with all other groups by one-way ANOVA followed by Dunnett's multiple comparison test. Differences were judged as significant if the *p*-value was <0.05.

## Results

### Metabolic activity and integrity of HLC in 2D cultures or 3D bioreactors

**FIG. 1.**
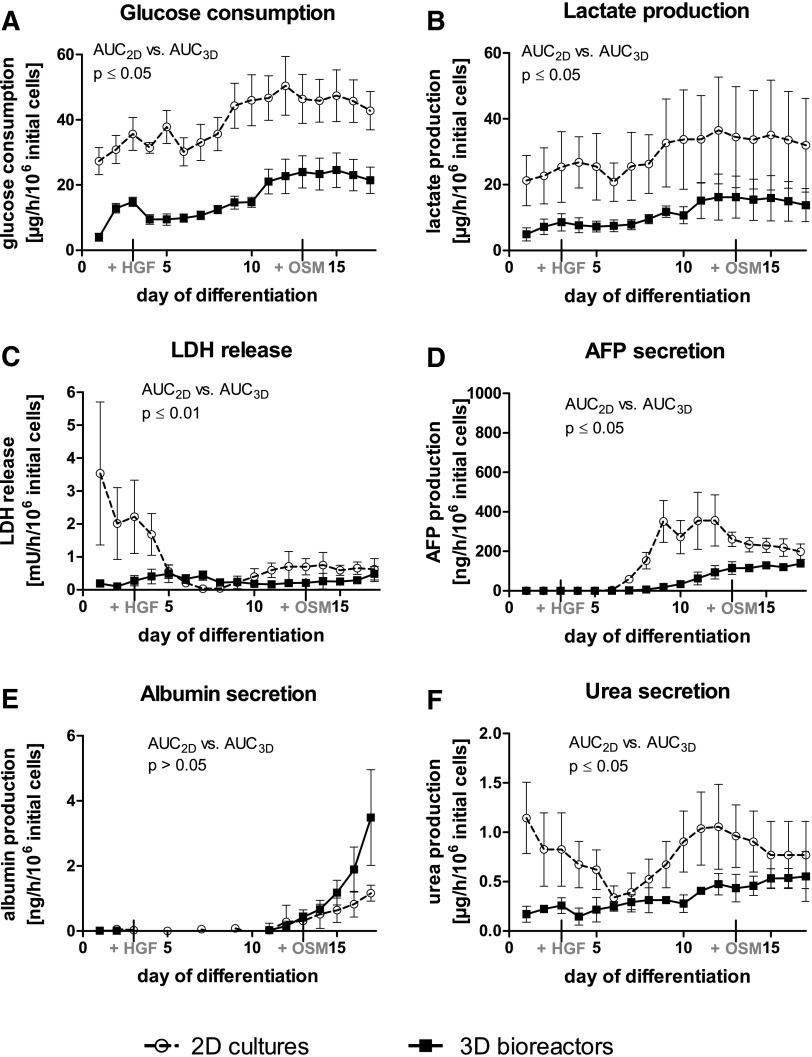
Metabolic activity of hiPSC during hepatic differentiation in 2D cultures (dotted line) or in 3D bioreactor cultures (black line). **(A)** Glucose consumption, **(B)** lactate production, **(C)** release of LDH, **(D)** secretion of AFP, **(E)** albumin production, and **(F)** urea secretion. Values were normalized to 1 × 10^6^ inoculated cells. AUC was calculated and differences were detected with the unpaired, two-tailed Student's *t*-test (3D bioreactors: *n* = 3, 2D cultures: *n* = 4, mean ± SEM). AFP, alpha-fetoprotein; AUC, area under curve; 2D, two dimensional; 3D, three dimensional; hiPSC, human induced pluripotent stem cells; LDH, lactate dehydrogenase; SEM, standard error of the mean.

In both culture systems, the metabolic activity of the cells during hepatic differentiation, assessed by glucose consumption and lactate production, showed an increase over time (Fig. 1A, B). Average glucose consumption rates were significantly higher (*p* ≤ 0.05) in 2D cultures than in 3D bioreactors (Fig. 1A). The time course of lactate production reflected that of glucose consumption (Fig. 1B). LDH release, indicating cell injury, was significantly higher (*p* ≤ 0.01) in 2D cultures than in 3D bioreactors during the first 3 days of differentiation, but decreased from day 3 on to basal levels. In contrast, LDH release in 3D bioreactors remained on a basal level over the whole culture period (Fig. 1C).

### Secretion of stage-specific proteins and metabolites

The synthesis of liver-specific proteins and the activity of the urea cycle were evaluated during hepatic differentiation. The endoderm-specific marker AFP showed an increase during the hepatoblast differentiation stage, starting on day 7 in 2D cultures and on day 9 in 3D bioreactors, but the increase was significantly higher (*p* ≤ 0.05) in 2D cultures (Fig. 1D). Albumin also showed an increase during the hepatoblast differentiation phase beginning on day 12 of differentiation in 2D cultures and 3D bioreactors. However, maximum values achieved were thrice higher in 3D bioreactors than in 2D cultures (Fig. 1E), although the difference was not significant.

Values of urea secretion showed a constant increase in 3D bioreactors until day 17 to the threefold of secretion rates detected on day 1 of culture. A fluctuating time course was observed in 2D cultures with peaks at the beginning of culture and between day 9 and 13 (Fig. 1F). Values approximated those of 3D bioreactors during the final days of differentiation.

### Functional analysis of different cytochrome P450 (CYP) isoenzymes

To investigate the CYP activity, the ability of the HLC to metabolize various substrates into their isoenzyme-specific products was analyzed and compared with CYP activities of undifferentiated hiPSC and those of PHH cultures determined 24 h after seeding.

**FIG. 2.**
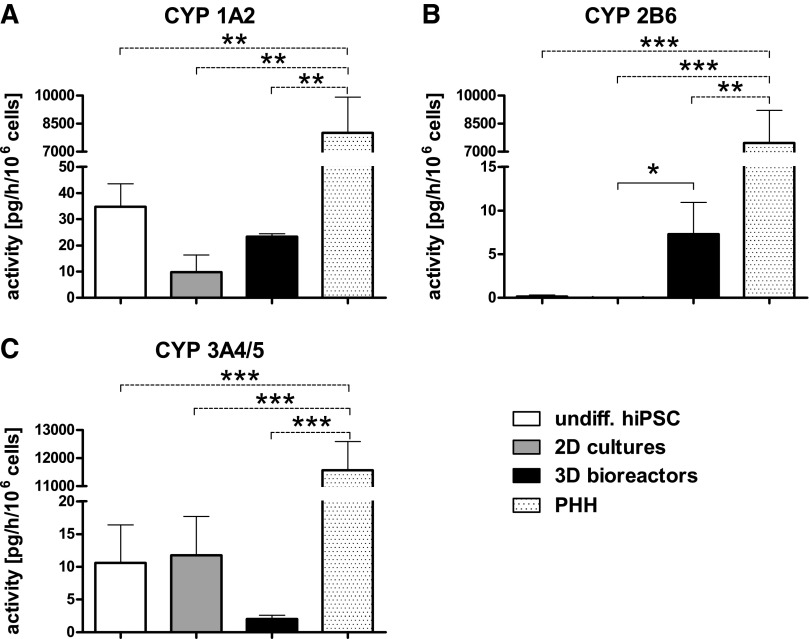
Activities of different cytochrome P450 (CYP) isoenzymes in undifferentiated hiPSC (white), in hiPSC after hepatic differentiation in 2D cultures (gray) or 3D bioreactors (black), or in PHH (dotted). CYP activities were determined by measuring the conversion rates of selected substrates into isoenzyme-specific products. **(A)** Formation of acetaminophen from phenacetin by CYP1A2, **(B)** formation of 6-OH-bupropion from bupropion by CYP2B6, and **(C)** formation of 1-OH-midazolam from midazolam by CYP3A4/5. Differences in metabolic activity between undifferentiated hiPSC, 2D cultures and 3D bioreactors, were calculated using one-way ANOVA with Bonferroni's multiple comparison test (solid line). In addition, differences between PHH to all other groups were calculated using one-way ANOVA with Dunnett's multiple comparison test (dotted line). (3D bioreactors: *n* = 3, 2D cultures and undifferentiated hiPSC: *n* = 4, PHH: *n* = 5; mean ± SEM), *p ≤ 0.05, **p ≤ 0.01, ***p ≤ 0.001. ANOVA, analysis of variance; PHH, primary human hepatocytes.

CYP1A2 showed a higher activity in 3D bioreactors than in 2D cultures, while it was lower compared with undifferentiated hiPSC or PHH (Fig. 2A). A significant CYP2B6-dependent conversion of bupropion to 6-OH-bupropion was observed in 3D bioreactors compared to 2D cultures (*p* ≤ 0.05 Fig. 2B). In contrast, CYP3A4 showed higher activities in undifferentiated hiPSC and in 2D cultures than in 3D bioreactors, although the differences were not significant (Fig. 2C). The isoenzyme CYP2C9 activity was detected neither in differentiated nor in undifferentiated hiPSC (data not shown). All enzyme activities investigated were significantly higher in PHH than in the HLC generated from hiPSC in 2D or 3D cultures (Fig. 2A–C).

### Gene expression of pluripotency factors, and endodermal and hepatic markers

**FIG. 3.**
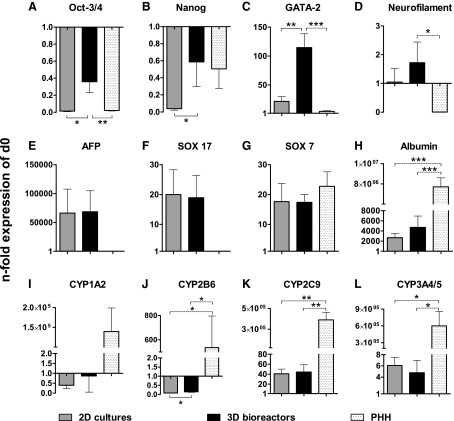
Gene expression of pluripotency markers **(A)** Oct-3/4 and **(B)** Nanog, mesodermal marker **(C)** GATA-2, ectodermal marker **(D)** neurofilament, endodermal markers **(E)** AFP and **(F)** SOX 17, extra-embryonic marker **(G)** SOX 7 and hepatic markers **(H)** albumin, **(I)** CYP1A2, **(J)** CYP2B6, **(K)** CYP2C9 and **(L)** CYP3A4/5 in hiPSC after hepatic differentiation in 2D cultures or 3D bioreactors, and in PHH relative to undifferentiated hiPSC (d0). Samples for mRNA expression analysis were taken after hepatic differentiation of hiPSC in 2D cultures (black) or 3D bioreactors (gray). For mRNA expression analysis of PHH (dotted), freshly isolated cells were used. Fold changes relative to undifferentiated hiPSC were calculated with normalization to GAPDH expression by the ΔΔCt method. Differences in gene expression between 2D cultures and 3D bioreactors were calculated using the unpaired, two-tailed Student's *t*-test (solid line). In addition, differences between PHH and all other groups were calculated by means of one-way ANOVA with Dunnett's multiple comparison test (dotted line) (3D bioreactors: *n* = 3, 2D cultures and undifferentiated hiPSC: *n* = 4, PHH: *n* = 3; mean ± SEM), *p ≤ 0.05, **p ≤ 0.01, ***p ≤ 0.001.

The expression of 12 stage-specific genes was comparatively analyzed by qRT-PCR in 2D or 3D cultures of HLC and in freshly isolated PHH (Fig. 3). Expression levels were plotted relative to those of undifferentiated hiPSC (d0) to investigate the hepatic maturation state of the cells.

The expression of the pluripotency markers *POU5F1* and *NANOG* decreased in both culture systems compared to undifferentiated hiPSC, but the decrease was significantly higher in 2D cultures (*p* ≤ 0.05, Fig. 3A, B). PHH showed *POU5F1* levels similar to HLC in 2D cultures, while *NANOG* expression was similar to those in 3D bioreactors (Fig. 3A, B).

To exclude differentiation into nonendodermal cells, markers for mesoderm (*GATA2*) and endoderm (neurofilament, *NEFL*) were analyzed as well. The analysis revealed a >100-fold increase for *GATA2* in 3D bioreactors, which is significantly higher than in 2D cultures showing an ∼20-fold increase (Fig. 3C). The expression of *NEFL* did not change significantly in comparison to undifferentiated hiPSC (Fig. 3D). PHH showed only scarce expression of *NEFL* and *GATA2* (Fig. 3C, D).

In contrast, most markers for endoderm and mature hepatocytes increased in both culture systems. *AFP* showed a >60,000-fold increased expression in both 2D cultures and 3D bioreactors, relative to undifferentiated hiPSC (Fig. 3E), and the expression of *SOX17* (Fig. 3F), another endodermal marker, showed a 20-fold increase, while PHH showed no relevant expression of those markers. Since *AFP* and *SOX17* are markers for both, definitive and extraembryonic endoderm, *SOX7* (Fig. 3G) was analyzed as a specific marker for extraembryonic endoderm. The expression of *SOX7* was increased in both culture systems and was similar to that detected in PHH.

Among the markers investigated for mature hepatocytes, the highest increase was detected for albumin (*ALB*), which was increased in both groups by >2,000-fold, with 3D bioreactors showing twice the expression compared to 2D cultures (Fig. 3H).

In contrast, the expression of *CYP1A2* was decreased compared with undifferentiated hiPSC both in 2D cultures and 3D bioreactors (Fig. 3I), which is in line with the results from activity measurements (Fig. 2A). A decrease of mRNA expression was also detected for *CYP2B6*, but the expression was still significantly higher in 3D bioreactors (*p* ≤ 0.05, Fig. 3J) and is in accordance to the findings from activity measurements (Fig. 2B). Expression of *CYP2C9* and *CYP3A4/5* was increased by around 40- or five-fold, respectively, compared with undifferentiated cells showing no significant differences between 2D cultures and 3D bioreactors (Fig. 3K, L). All markers for mature hepatocytes showed a distinctly lower expression in HLC than in PHH, irrespective of the culture system.

### Immunohistochemical characterization of hiPSC-derived differentiated cells

**FIG. 4.**
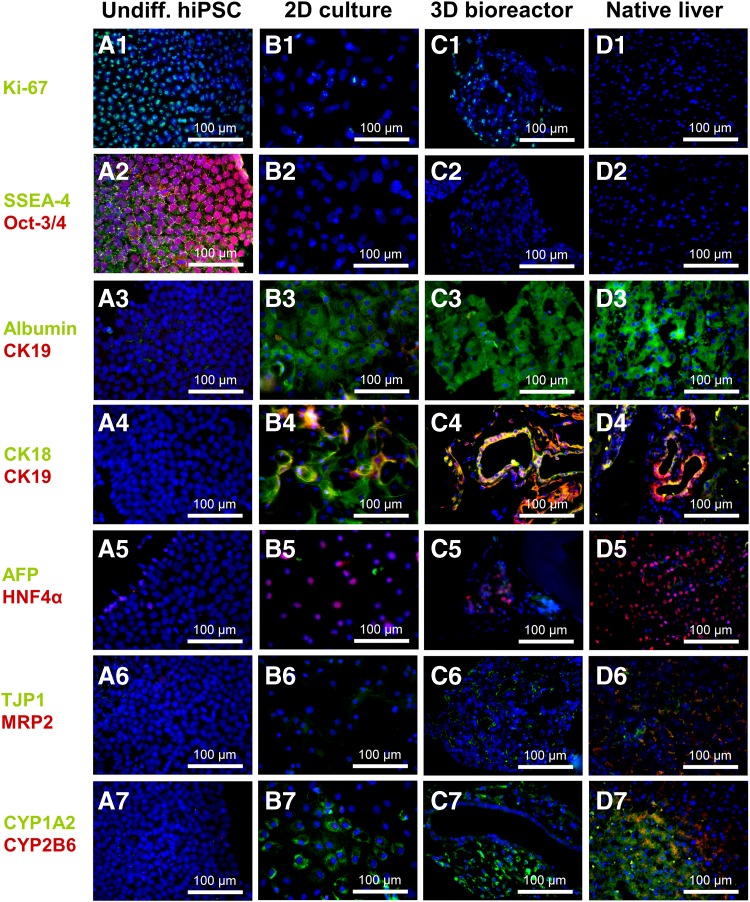
Immunofluorescence analysis of hiPSC before and after hepatic differentiation in 2D cultures or 3D bioreactors compared with native human liver tissue. Samples from cultures or liver tissue were stained with **(A1–D1)** Ki-67, **(A2–D2)** SSEA-4 and Oct-3/4, **(A3–D3)** albumin and CK19, **(A4–D4)** CK18 and CK19, **(A5–D5)** AFP and HNF4α, **(A6–D6)** TJP1 and MRP2, and with **(A7–D7)** CYP1A2 and CYP2B6. Nuclei were counterstained with DAPI (blue) or with bisBenzimide H 33342 trihydrochloride in native human liver tissue (blue). CK19, cytokeratin 19.

To determine the amount of proliferating cells before, during, and after the hepatic differentiation of hiPSC, an immunofluorescence staining with the proliferation marker Ki-67 was performed. In cultures of undifferentiated hiPSC, almost all cells (97.7% ± 1.6%) were positive for Ki-67 (Fig. 4A1). In 2D cultured DE cells, approximately two thirds of the cells (63.6% ± 12.6%) were proliferating (picture not shown). After hepatic differentiation, around one third of the cells were still proliferating in 2D cultures (32.6% ± 20.4%, Fig. 4B1) and 3D bioreactors (30.3% ± 18.7%, Fig. 4C1). In the native human liver, no Ki-67-positive cells could be detected (Fig. 4D1).

The pluripotency markers SSEA4 and OCT3 were expressed in the majority of undifferentiated hiPSC (Fig. 4A2), but could not be detected in hiPSC-derived HLC in 2D cultures (Fig. 4B2) or 3D bioreactors (Fig. 4C2), or in native human liver tissue (Fig. 4D2). Regarding the expression of hepatocyte-specific markers, the majority of cells was positive for albumin in 2D cultures as well as 3D bioreactors (Fig. 4B3, C3), similar to native human liver tissue (Fig. 4D3).

Cytokeratin 19 (CK19), characteristic for biliary cells, was only detected in a few sparsely distributed cells in 2D cultures (Fig. 4B4), whereas in 3D bioreactors, ring-shaped cell arrangements lined with cells positive for CK18 and CK19 were observed, indicating formation of bile duct-like tubular structures (Fig. 4C4) resembling those occurring in native human liver tissue (Fig. 4D4). Staining for hepatocyte nuclear factor 4-alpha (HNF4A) showed a few positive cells, which were negative for AFP in both 2D cultures and 3D bioreactors (Fig. 4B5, C5). This is in contrast to native human liver tissue where almost all cells showed immunoreactivity for HNF4A (Fig. 4D5).

Staining of tight junction protein (TJP1) and the transporter multidrug resistance-associated protein 2 (MRP2) revealed no immunoreactivity in 2D cultures (Fig. 4B6), while in 3D bioreactors (Fig. 4C6), abundant cells were positive for TJP1 as indicated by thin borders between the cells. In the native liver tissue, most of the cells were double positive for MRP2 and TJP1 (Fig. 4D6).

Immunofluorescence analysis of CYP isoenzymes showed that most of the cells in 2D cultures and 3D bioreactors were positive for CYP1A2, but negative for CYP2B6 (Fig. 4B7, C7), which is in line with the functional analysis showing higher activities for CYP1A2. In 3D bioreactors, the inner ring of tubular-like structures lacked CYP1A2 immunoreactivity indicating a zonation of the cells. In native human liver tissue, cells were positive for both CYP isoenzymes (Fig. 4D7). The undifferentiated hiPSC were negative for all liver-specific markers (Fig. 4A3–A7).

### Ultrastructural characteristics of hiPSC-derived differentiated cells

**FIG. 5.**
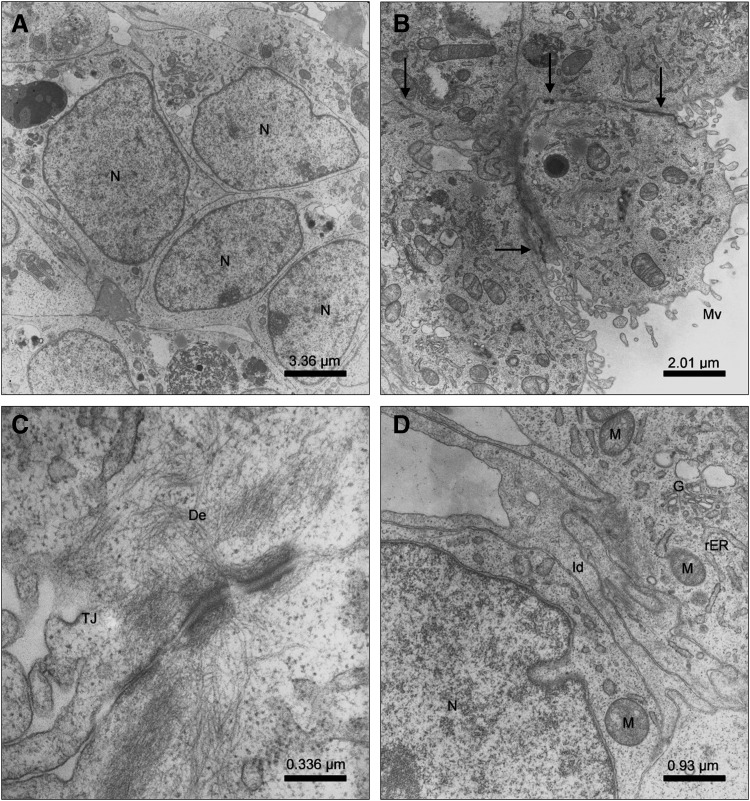
Ultrastructural characteristics of hiPSC after hepatic differentiation in 3D bioreactors. **(A)** Cells with a high nucleus:cytoplasm ratio indicating immature cells (N). **(B)** Cells with distinct microvilli (Mv) and abundant cell–cell contacts (arrows) between neighboring cells. **(C)** Tight junction (TJ) and desmosome (De) between two neighboring cells. **(D)** Interdigitations (Id) between two cells with mitochondria (M), rough endoplasmic reticulum (rER), and Golgi apparatus (G).

Ultrastructural studies of hiPSC-derived cells after hepatic differentiation in 3D bioreactors showed a heterogeneous cell population with some similarities to native liver tissue. Cells displaying a high nucleus to cytoplasm ratio indicate the presence of still immature cells (Fig. 5A). In other areas, cells showed microvilli at their apical side with abundant cell–cell contacts consisting of tight junctions and desmosomes indicating cell polarization typical for hepatocytes (Fig. 5B, C). The majority of cells contained numerous mitochondria, which is also a typical feature of hepatocytes due to their high metabolic activity. Distinct interdigitations between neighboring cells and rough endoplasmic reticulum in the cytoplasm were observed in addition to well-developed Golgi apparatuses (Fig. 5D).

## Discussion

Hepatocytes derived from hiPSC represent a promising cell source for pharmacological toxicity testing. Although numerous protocols for the hepatic differentiation of hiPSC exist and are continuously refined, the obtained cells still show a fetal phenotype,^16,17^ and the maturation state of the obtained cells needs to be further improved. In this study, we investigated the potency of a dynamic 3D bioreactor technology to promote the hepatic maturation of hiPSC-derived hepatocytes when compared to conventional 2D cultures. The differentiation outcome of hiPSC cultured in 2D cultures or 3D bioreactors was analyzed by means of metabolic, phenotypical, and functional parameters.

To allow a direct comparison of the two culture systems, the results for secreted metabolites and proteins as well as for CYP activities were normalized to the initial cell number. Potential proliferation activities of HLC in 2D cultures or 3D bioreactors were quantified by means of Ki-67 staining. The protein Ki-67 is expressed during all active phases of the cell cycle (G1, S, G2, mitosis), but is absent in resting cells (G0).^35^ The quantitative analysis of the Ki-67 staining revealed that at the end of hepatic differentiation, there was still mitotic activity. However, since the amount of proliferating cells with ∼30% was comparable in 2D cultures and 3D bioreactors, it can be concluded that the observed differences in metabolite/protein secretion and CYP activities are not associated with differences in cell growth.

Glucose consumption rates and lactate production rates as indicators for energy metabolism were doubled in 2D cultures compared to 3D bioreactors. This could be caused by the formation of 3D cell aggregates within the bioreactor leading to a gradient and a differential metabolic activity of cells growing either on the surface or in the center of the aggregates. It has been reported that concentration gradients influence cell differentiation and tissue formation.^36,37^ Thus, formation of gradients could also be a reason for the heterogeneous cell population observed in 3D bioreactors as indicated by immunohistochemical analyses and increased *GATA2* mRNA expression.

In the described hollow fiber bioreactor, the aggregate size is controlled by the distance between the capillaries. In other bioreactor technologies such as stirred tank vessels, the aggregate size is influenced by the impeller design^38^ and the stirring velocity,^39^ but stirring can cause shear stress and cell damage.^40^ Another bioreactor technology with minimized shear stress is the rotating-wall vessel, which consists of a horizontally rotating cylindrical culture vessel with a coaxial tubular oxygenator avoiding nutrient compartmentalization and barrier formation.^41^

Furthermore, a significantly lower LDH release was detected in 3D bioreactors compared with 2D cultures. One possible reason could be a protective effect of 3D cell aggregates against damage caused by the applied activin A, which can induce apoptosis.^42^ However, LDH is released during both necrosis and apoptosis^43^ and therefore does not allow distinguishing between necrosis and apoptosis. An apoptosis assay would be needed to clarify whether the observed LDH release is due to apoptosis processes.

AFP expression and secretion were detected in HLC in both 2D cultures and 3D bioreactors, with significantly higher secretion rates in 2D cultures. In contrast, albumin secretion showed a distinctly higher increase in 3D cultures than in 2D cultures. *In vivo* AFP is only expressed during liver embryogenesis, in fetal liver cells, and hepatocellular carcinomas,^44^ while albumin is produced by differentiated hepatocytes in the adult liver. Thus, the findings of *AFP* and *ALB* expression indicate a higher maturation grade of HLC in 3D bioreactors than in 2D cultures.

Expression of *AFP* in HLC in association with expression of markers for mature hepatocytes such as *ALB* and various *CYP* isoenzymes has been described in several studies.^7,45,46^ Gieseck et al.^47^ also observed lower AFP secretion rates and higher albumin secretion rates in 3D clump cultures compared to 2D cultures, which emphasizes the value of 3D culture systems to promote hepatic maturation of hiPSC. Since albumin secretion was still increasing at the end of hepatic differentiation, a further maturation could be achieved by prolongation of the differentiation period.

This finding is supported by the observed increase of urea secretion in 3D bioreactors. Urea is the major end product of protein nitrogen metabolism and is synthesized by the urea cycle in the liver from ammonia. Hence, urea secretion is an important functional marker for mature hepatocytes. To further validate results from urea secretion, the expression of enzymes from the urea cycle such as carbamoyl phosphate synthetase I or transcarbamylase could be analyzed.

In addition to *AFP* and *SOX17* as markers for both, definitive and extraembryonic endoderm, *SOX7* was analyzed as a specific marker for extraembryonic endoderm. The gene expression of *SOX7* was similar in HLC in 2D cultures, in 3D bioreactors, and in PHH, indicating that the hiPSC-derived HLC did not contain significant amounts of extraembryonic cells, which confirms the hepatic origin of *AFP* and also of *SOX17* in this study. According to the Human Protein Atlas, the *SOX7* expression is strongest in the placenta, but a weak expression is also found in the human liver.^48^

The ability to metabolize drugs through specific CYP isoenzymes is crucial for potential application of hiPSC-derived HLC in pharmacological studies. Functional activities of CYP1A2 and CYP3A4 were detected in both 2D cultures and 3D bioreactors, while the CYP2B6 activity was observed only in 3D bioreactors. However, all CYP isoenzyme activities were significantly lower in HLC than in PHH. Data from other studies also showed a lower expression and activity of CYPs in differentiated hiPSC compared to PHH.^5,49^ However, since different experimental conditions were used in those studies, a direct comparison of CYP activities from different publications is difficult.

Measurements of mRNA expression levels revealed that the pluripotency markers *POU5F1* and *NANOG* were stronger downregulated in 2D cultures than in 3D bioreactors. This could be explained by the formation of an activin A gradient within the 3D cell aggregates. Activin A recapitulates the nodal signaling pathway *in vitro*, which stimulates DE derivation from pluripotent stem cells.^50^ In lower concentrations, activin A is used to maintain pluripotency of hiPSC^51^ and hESC.^52^ This could imply that DE formation is decreased in the center of the cell aggregates and other factors such as dimethylsulfoxide (DMSO) might be required to block the effect of activin A on the pluripotency.^53^

On the other hand, the formation of gradients of activin A and other factors might also promote 3D tissue formation from HLC by reflecting the physiological situation in the liver lobule, which is characterized by an oxygen gradient between the periportal and the perivenous area. This is supported by immunohistochemical and TEM pictures showing tissue-like organization of the cells within 3D bioreactors, including bile duct-like structures characterized by CK18/CK19 double staining. Miki et al.^26^ also observed the formation of bile duct-like structures after hepatic differentiation of hESC in the 3D bioreactor. Bile ducts are lined with cholangiocytes, which, like the hepatocytes, originate from the bipotent hepatoblasts.^54^

Current protocols for direct differentiation of pluripotent stem cells to cholangiocytes and their *in vitro* generation from hepatoblasts are to some extent similar to protocols used for hepatocyte differentiation since they use factors such as epidermal growth factor, insulin, and hydrocortisone.^55,56^ De Assuncao et al.^55^ observed that cholangiocyte markers already became apparent during the hepatic progenitor phase, which would explain the appearance of cells positive for CK19 in this study.

In addition, abundant tight junctions were detected by immunohistochemical staining and ultrastructural analysis using TEM. The presence of tight junctions is a prerequisite for the formation of bile canaliculi as a characteristic feature of differentiated hepatocytes. In contrast to tight junction protein, the transporter protein MRP2, which is expressed in the canalicular (apical) membrane area of the hepatocyte and contributes to biliary transport, was negative both in 2D cultures and 3D bioreactor cultures, indicating incomplete cell polarization.

The detailed comparison of hiPSC-derived HLC with PHH showed that further improvement of HLC maturation is needed. This could be achieved by combining other promising differentiation strategies with the 3D bioreactor culture: for example, differentiation into the mesodermal direction during DE differentiation, detected by *GATA2* expression in the 3D bioreactor, could be excluded by adding specific inhibitors^57^ and cocultivation with primitive endothelial cells or mesenchymal stem cells could recapitulate the *in vivo* organogenesis even more closely in the 3D culture.^15^ In addition, Kim et al.^46^ could show that repeated stimulation of HLC with xenobiotics could improve their metabolizing activity.

A further strategy is based on the usage of an hiPSC line, which is derived from primary hepatoblasts as already shown for mouse iPSC.^58^ This could also improve the hepatic differentiation since recent studies suggest that iPSC retain a residual donor cell memory, which may impact their capacity to differentiate into the cell type of origin.^59^

## Conclusion

In conclusion, the hepatic maturation of hiPSC-derived HLC was improved in 3D bioreactors compared with 2D cultures in terms of albumin secretion, CYP2B6 activity, and formation of tissue-like structures with cell–cell contacts. However, some aspects of hepatic functionality were still insufficient, for example, *AFP* was still expressed, and the activity of CYP2C9 and expression of the biliary transporter MRP2 could not be detected. The 3D bioreactor could be used to investigate approaches to improve the maturation of hiPSC-derived hepatocytes and generate fully differentiated hepatocytes from hiPSC in a physiological-like 3D environment in the future.

## Abbreviations Used

ANOVA: analysis of variance
AUC: area under curve
DE: definitive endoderm
hESC: human embryonic stem cells
HGF: hepatocyte growth factor
hiPSC: human induced pluripotent stem cells
HLC: hepatocyte-like cells
LDH: lactate dehydrogenase
PHH: primary human hepatocytes
TEM: transmission electron microscopy
